# Acceptance, completion, and safety of the 3HR regimen for latent tuberculosis infection: a prospective cohort study in China

**DOI:** 10.3389/fpubh.2026.1794409

**Published:** 2026-04-20

**Authors:** Qingfeng Sun, Kai Zhang, Jifei Chen, Lizhen Feng, Liuchun Shi, Sang Liu, Aimei Liu

**Affiliations:** 1 Department of Tuberculosis, Guangxi Zhuang Autonomous Region Chest Hospital, Liuzhou, Guangxi, China; 2 Department of Public Health, Guangxi Zhuang Autonomous Region Chest Hospital, Liuzhou, Guangxi, China; 3 Department of Science and Education, Guangxi Zhuang Autonomous Region Chest Hospital, Liuzhou, Guangxi, China

**Keywords:** 3HR regimen, acceptance and completion, adverse events, latent tuberculosis infection, prospective cohort study

## Abstract

**Background:**

Tuberculosis preventive treatment (TPT) is essential for reducing the progression of latent tuberculosis infection (LTBI) to active disease, yet real-world implementation often faces substantial attrition. This study evaluated the acceptance, completion, and safety of the 3-month daily isoniazid plus rifampicin regimen (3HR) among close contacts in a high-burden setting in China.

**Methods:**

We conducted a prospective observational cohort study among tuberculin skin test–positive close contacts from January 2024 to June 2025. Participants voluntarily chose to initiate or decline 3HR. Acceptance, completion, and adverse events (AEs) were prospectively recorded. Multivariable logistic regression models were used to identify predictors of uptake and completion, and a sensitivity analysis assessed the differential impact of hepatic versus non-hepatic AEs.

**Results:**

Among 617 eligible contacts, 520 (84.3%) accepted TPT. Acceptance was significantly higher among school than household contacts (96.0% vs. 76.1%; *p* < 0.001), and school contact status was the strongest predictor of uptake (adjusted odds ratio [aOR] 14.20; 95% CI 5.48–36.82). Among the 520 initiators, 382 (73.5%) completed treatment. Demographic factors were not associated with completion, whereas AEs markedly reduced completion (aOR 0.25; 95% CI 0.14–0.44). A total of 56 participants (10.8%) experienced at least one AE; hepatic events were the most frequent (73.2%) and accounted for most discontinuations. Subjective intolerance far exceeded clinically confirmed AEs (75 vs. 20). Sensitivity analyses confirmed that both hepatic and non-hepatic AEs independently reduced completion.

**Conclusion:**

In this real-world implementation evaluation, the 3HR regimen demonstrated high programmatic acceptance, particularly in school settings; however, treatment completion was constrained by both perceived and clinically verified AEs. These findings suggest that optimizing TPT delivery requires strengthened safety monitoring, structured symptom counseling, and targeted mobilization strategies to improve uptake, particularly among household contacts.

## Introduction

Tuberculosis (TB) remains the world’s leading cause of death from a single infectious agent, despite being a preventable and curable disease. According to the WHO Global Tuberculosis Report 2025, an estimated 10.7 million people developed TB in 2024, reversing the COVID-19–related surge observed between 2021 and 2023 but still exceeding pre-pandemic levels (1). China accounts for approximately 6.5% of global incident cases, ranking among the eight countries that together contribute two-thirds of the global TB burden. Although incidence and mortality rates have begun to decline, global progress remains far off track from the End TB Strategy milestones, which require a 50% reduction in incidence and a 75% reduction in TB deaths by 2025 compared with 2015 baselines (2).

A major obstacle to TB elimination is the enormous reservoir of tuberculosis infection, affecting roughly one-quarter of the world’s population (3). Following infection, the risk of progression to active disease is highest in the first 2 years, during which approximately 5% develop TB (4). This continual flux from latent infection to active disease sustains ongoing transmission and underpins the global epidemic. Consequently, programmatic management of TB infection, particularly tuberculosis preventive treatment (TPT) for high-risk populations such as household contacts, has become central to modern TB control efforts (5). However, implementation gaps persist. In 2024, global TPT coverage reached only 25% among household contacts, falling significantly short of the United Nations target of 90% coverage by 2027 (1).

To bridge this gap and improve uptake, treatment paradigms are shifting away from the traditional 6–9 months of daily isoniazid monotherapy (6H/9H), which is often plagued by poor adherence and hepatotoxicity concerns (6, 7). WHO now prioritizes shorter, rifamycin-based regimens that offer non-inferior efficacy with higher completion rates (5–8). Among these, the 3-month regimen of daily isoniazid and rifampicin (3HR) has emerged as a pragmatic alternative (5). Unlike the weekly isoniazid-rifapentine (3HP) regimen, which faces supply chain constraints in some regions, 3HR utilizes first-line drugs that are widely available and affordable in many low- and middle-income countries, facilitating its integration into existing national TB programs (9, 10).

Despite the availability of shorter regimens, effective program performance is not guaranteed. In China, recent national guidance has prioritized TPT for close contacts (11), yet the scale-up faces significant challenges regarding safety concerns and population acceptance (12). Clinical apprehension regarding drug-induced liver injury (DILI) remains a major barrier to prescribing TPT, particularly for older adults (13), while the asymptomatic nature of infection often leads to a low perception of risk among contacts (14). Furthermore, TB control in China faces a dual challenge: managing sporadic transmission in households and controlling clustered outbreaks in schools (15). Few studies have prospectively compared the TPT cascade between these distinct settings, namely schools versus households, where social dynamics differ fundamentally. Additionally, most existing studies focus solely on patients who initiate treatment, failing to capture the characteristics of the “refusal population,” which is essential for designing targeted mobilization strategies.

To address these knowledge gaps, we conducted a prospective cohort study in a high-burden setting in China. By offering the 3HR regimen to eligible close contacts and establishing a non-intervention control group for those who declined, we aimed to describe real-world programmatic acceptance and completion rates and systematically characterize the safety profile, particularly hepatotoxicity and its impact on discontinuation. Additionally, this study sought to identify independent predictors of treatment uptake and adherence. Through a comparative analysis of household and school contacts, we provide evidence-based insights to optimize TPT delivery strategies.

## Methods

### Study design and setting

This was a prospective observational cohort study designed as a real-world implementation evaluation of TPT among close contacts with latent tuberculosis infection (LTBI) at Guangxi Zhuang Autonomous Region Chest Hospital, China, from January 2024 to June 2025. Participants voluntarily chose whether to initiate the 3HR regimen. The study protocol was approved by the Ethics Committee of Guangxi Zhuang Autonomous Region Chest Hospital (IRB Approval No. 2021–011-03), and was carried out in accordance with the principles of the Declaration of Helsinki. Written informed consent was obtained from all participants or their legal guardians prior to enrollment. As this was a non-interventional observational study conducted as a real-world implementation evaluation, prospective clinical trial registration was not required.

### Participants and LTBI screening

Close contacts were identified through routine household and school-based contact investigations (5, 15). Latent tuberculosis infection (LTBI) screening was performed using the tuberculin skin test (TST) in accordance with Chinese National TB Control Programme guidance and the 2024 WHO consolidated guidelines on tuberculosis preventive treatment (5). A positive TST result was defined as an induration diameter ≥10 mm. Because China implements universal BCG vaccination at birth, this threshold was applied consistently in accordance with national guidance; IGRA was not routinely available at our center during the study period. Individuals were eligible for inclusion if they had documented exposure to an index pulmonary tuberculosis case, a positive TST result, and no clinical or radiographic evidence of active tuberculosis (16). Participants were also required to be clinically suitable for preventive therapy and willing to participate in the study. Individuals were excluded if they had active tuberculosis disease; contraindications to isoniazid or rifampicin; significant hepatic, renal, hematologic, or neurological conditions; a history of tuberculosis preventive therapy within the preceding 3–5 years; pregnancy or lactation; or any condition that a clinician considered inappropriate for preventive treatment.

### Intervention and procedures

All index pulmonary tuberculosis cases had bacteriologically confirmed disease and underwent drug susceptibility testing by phenotypic methods and/or Xpert MTB/RIF. All were confirmed to be susceptible to both isoniazid and rifampicin, and no contacts of index cases with confirmed or presumptive drug-resistant tuberculosis were enrolled. Participants who accepted preventive treatment received a standardized 3HR regimen consisting of isoniazid 300 mg once daily plus rifampicin dosed by body weight (450 mg daily for <50 kg and 600 mg daily for ≥50 kg) (5). After counseling regarding the benefits and risks of treatment, participants either initiated the 3HR regimen (intervention group) or declined preventive therapy (non-intervention group). Those who initiated treatment were followed at Weeks 4, 8, and 12 to assess adherence, monitor adverse events, and perform laboratory testing. Laboratory assessments, including liver function tests, complete blood count, and urinalysis, were obtained at baseline and at each scheduled follow-up visit, with additional assessments performed when prompted by patient-reported symptoms. Those who declined treatment received routine health education and observation.

### Data collection and outcome measures

Baseline demographic and clinical information was collected for all enrolled participants, including age, sex, body mass index (BMI), type of contact exposure (household vs. school), residence (urban vs. rural), and behavioral factors. Smoking history was defined as having smoked at least 100 cigarettes in one’s lifetime; for regression analyses, smoking status was dichotomized as ever-smoker (current or former) versus never-smoker. Alcohol consumption was defined as any self-reported alcohol consumption within the past 12 months (yes versus no) (16). For participants who initiated TPT, treatment completion, reasons for discontinuation, adverse events, and laboratory monitoring results were recorded prospectively. The primary outcomes were TPT acceptance and treatment completion. Secondary outcomes included the incidence of adverse events and reasons for discontinuation. Discontinuation was classified as due to clinically confirmed adverse events, subjective intolerance, loss to follow-up, or voluntary withdrawal, with each event assigned one primary reason by the treating physician at the time of withdrawal.

### Adverse event classification

All AEs occurring during treatment were recorded prospectively at each scheduled follow-up visit (Weeks 4, 8, and 12) and at any unscheduled visit prompted by patient-reported symptoms. Events were classified as hepatic, gastrointestinal, skin-related, hematologic, respiratory, or constitutional. Clinically significant hepatotoxicity was defined according to American Thoracic Society criteria as alanine aminotransferase (ALT) elevation ≥3 times the upper limit of normal (ULN) in the presence of hepatitis symptoms and/or jaundice, or ALT elevation ≥5 times the ULN in the absence of symptoms; aspartate aminotransferase (AST) and total bilirubin were considered adjunctive markers in clinical assessment (17). Hepatic adverse events were graded according to WHO toxicity criteria (grades 1–4), with Grade 1–2 considered mild to moderate and Grade 3–4 severe. Treatment was withheld when hepatotoxicity met ATS interruption criteria, and reinitiation or permanent discontinuation was determined according to clinical and biochemical recovery. Non-hepatic AEs were managed individually, with temporary interruption or permanent discontinuation according to clinical severity. For this study, subjective intolerance was operationally defined as treatment discontinuation prompted by patient-reported symptoms or concern about side effects in the absence of objectively verifiable evidence of clinically significant toxicity. Cases were classified as subjective intolerance when patient-reported symptoms were not accompanied by clinical or laboratory findings meeting the prespecified criteria for a clinically significant adverse event, and the treating senior clinician judged that the primary reason for discontinuation was symptom burden without objective confirmation of a treatment-limiting adverse drug reaction. All such determinations were made prospectively at the time of discontinuation and documented in standardized case report forms.

### Statistical analysis

Variables included in the multivariable models were prespecified *a priori* based on prior literature, clinical relevance, and variable availability with sufficient completeness to support stable estimation, rather than selected using data-driven procedures. Continuous variables were summarized as medians and interquartile ranges (IQRs) and compared using the Mann–Whitney *U* test, while categorical variables were presented as frequencies and percentages and compared using the chi-square test or Fisher’s exact test, as appropriate. Multivariable logistic regression was used to identify factors associated with TPT acceptance and treatment completion. The acceptance model included age, sex, contact type, BMI, residence, and smoking and drinking history, whereas the completion model included age, sex, contact type, BMI, residence, and occurrence of any adverse event; age and BMI were entered as continuous variables. Results were reported as adjusted odds ratios (aORs) with 95% confidence intervals (CIs). Missing data were handled using complete-case analysis, and no imputation was performed. The acceptance model was fitted using 518 participants with complete covariate data, and the completion model was fitted using 464 participants with complete covariate data. Acceptance status was available for all 617 eligible participants, and completion status was available for all 520 participants who initiated treatment. As this was a descriptive, real-world implementation evaluation, no formal *a priori* power calculation was performed; instead, all consecutive eligible close contacts identified through routine contact investigation at our center during the 18-month study period were included (*N* = 617). Sensitivity analyses were performed to assess whether hepatic and non-hepatic adverse events had differential associations with treatment completion. All statistical analyses were performed using Python (version 3.11.7). Logistic regression models were fitted using the statsmodels library (version 0.14.0), built on the SciPy scientific computing framework (18). Data management was conducted using pandas (version 2.1.4) and NumPy (version 1.26.4) (19), and figures were generated using matplotlib (version 3.8.0). All tests were two-sided, and *p* < 0.05 was considered statistically significant.

## Results

### Study population and baseline characteristics

**Figure 1:**
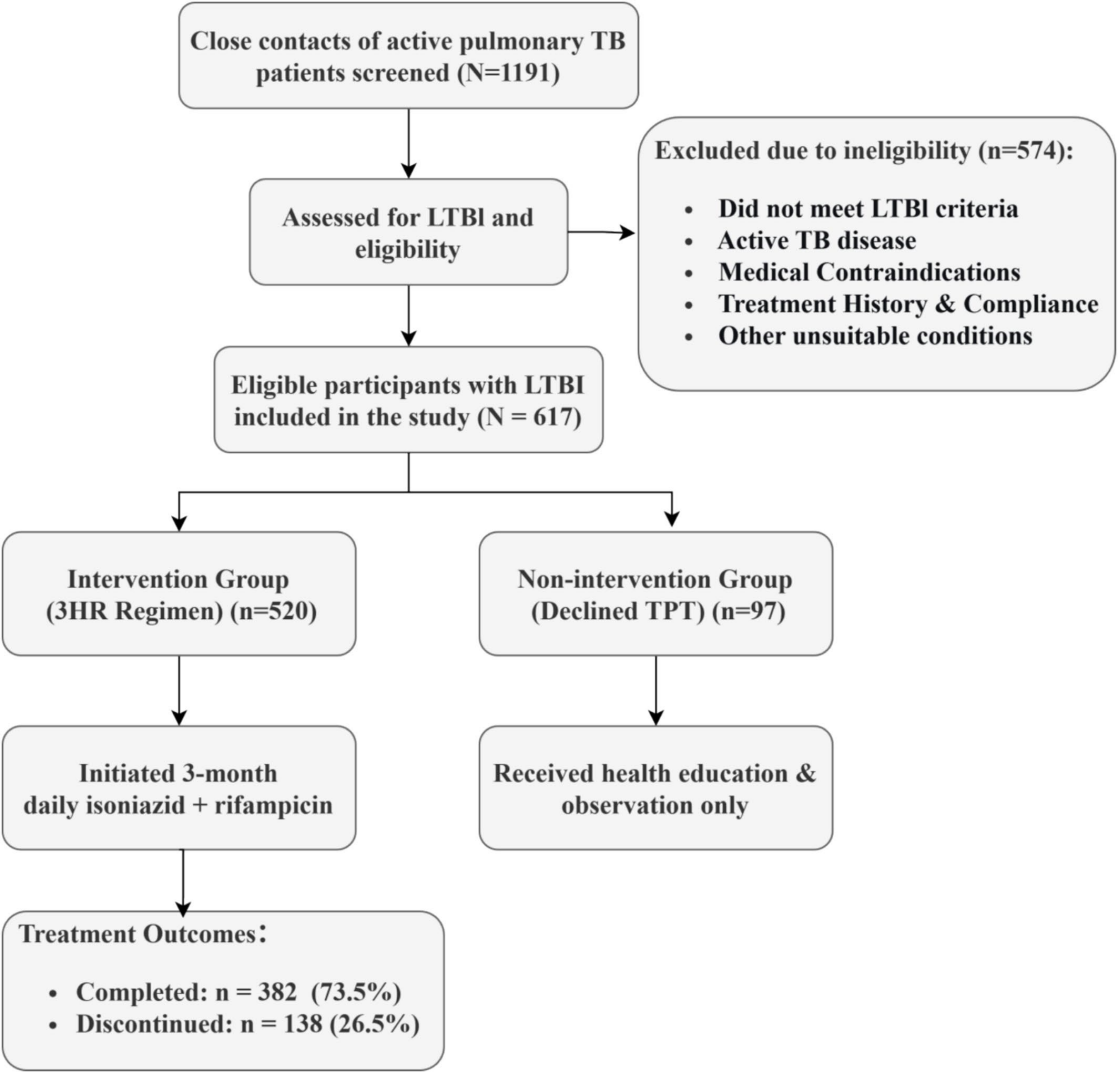
Study profile and TPT cascade flowchart.

A total of 1,191 close contacts of active pulmonary TB patients were screened. After excluding 574 individuals who did not meet eligibility criteria, 617 eligible participants with LTBI were included in the study (Figure 1). The overall median age was 21.5 years (IQR 17.0–45.0). Among these, 520 participants (84.3%) accepted and initiated the 3-month daily isoniazid plus rifampicin regimen, whereas 97 participants (15.7%) declined.

Participants who accepted TPT were significantly younger (median 19.0 vs. 37.0 years, *p* < 0.001) and more likely to be school contacts (46.7% vs. 10.3%, *p* < 0.001). Acceptance was also higher among participants residing in urban areas (*p* = 0.017) and in those without a history of alcohol consumption (*p* = 0.011). Sex, BMI, and smoking history did not differ significantly between groups (Table 1).

**Table 1:** Baseline demographic and clinical characteristics of participants stratified by TPT acceptance status.

Characteristics	Total (*N* = 617)	Accepted (*N* = 520)	Not accepted (*N* = 97)	*p*-value
Age (years), median [IQR]	21.5 [17.0–45.0]	19.0 [16.0–44.0]	37.0 [21.0–48.0]	**<0.001**
Age group, *n* (%)				<0.001
<15 years	60 (9.7%)	53 (10.2%)	7 (7.2%)	
15–25 years	274 (44.4%)	249 (47.9%)	25 (25.8%)	
>25 years	283 (45.9%)	218 (41.9%)	65 (67.0%)	
Sex, *n* (%)				0.915
Male	280 (45.4%)	235 (45.2%)	45 (46.4%)	
Female	337 (54.6%)	285 (54.8%)	52 (53.6%)	
Contact type, *n* (%)				<0.001
Household	364 (59.0%)	277 (53.3%)	87 (89.7%)	
School	253 (41.0%)	243 (46.7%)	10 (10.3%)	
BMI (kg/m^2^), median [IQR]	21.3 [19.1–24.6]	21.3 [19.1–24.5]	21.8 [19.6–25.2]	0.313
BMI category, *n* (%)^†^				0.425
Underweight (<18.5)	102 (18.3%)	87 (18.1%)	15 (19.5%)	
Normal (18.5–23.9)	290 (52.1%)	255 (53.1%)	35 (45.5%)	
Overweight/Obese (≥24)	165 (29.6%)	138 (28.8%)	27 (35.1%)	
Residence, *n* (%)^†^				0.017
Urban	377 (63.6%)	328 (65.7%)	49 (52.1%)	
Rural	216 (36.4%)	171 (34.3%)	45 (47.9%)	
Smoking history, *n* (%)^†^				0.264
Yes	95 (17.1%)	77 (16.2%)	18 (22.0%)	
No	462 (82.9%)	398 (83.8%)	64 (78.0%)	
Drinking history, *n* (%)^†^				0.011
Yes	135 (24.2%)	105 (22.1%)	30 (35.7%)	
No	424 (75.8%)	370 (77.9%)	54 (64.3%)	

^†^Variables with missing data. The number of participants with missing data: BMI category, *n* = 60 (accepted *n* = 40, not accepted *n* = 20); residence, *n* = 24 (accepted *n* = 21, not accepted *n* = 3); smoking history, *n* = 60 (accepted *n* = 45, not accepted *n* = 15); drinking history, *n* = 58 (accepted *n* = 45, not accepted *n* = 13). Percentages for these variables are calculated using the number of participants with available data in each group as the denominator.

IQR, interquartile range; BMI, body mass index; TPT, tuberculosis preventive treatment.

Bold values indicate statistical significance (*p* < 0.05).

### Predictors of TPT acceptance

**Figure 2:**
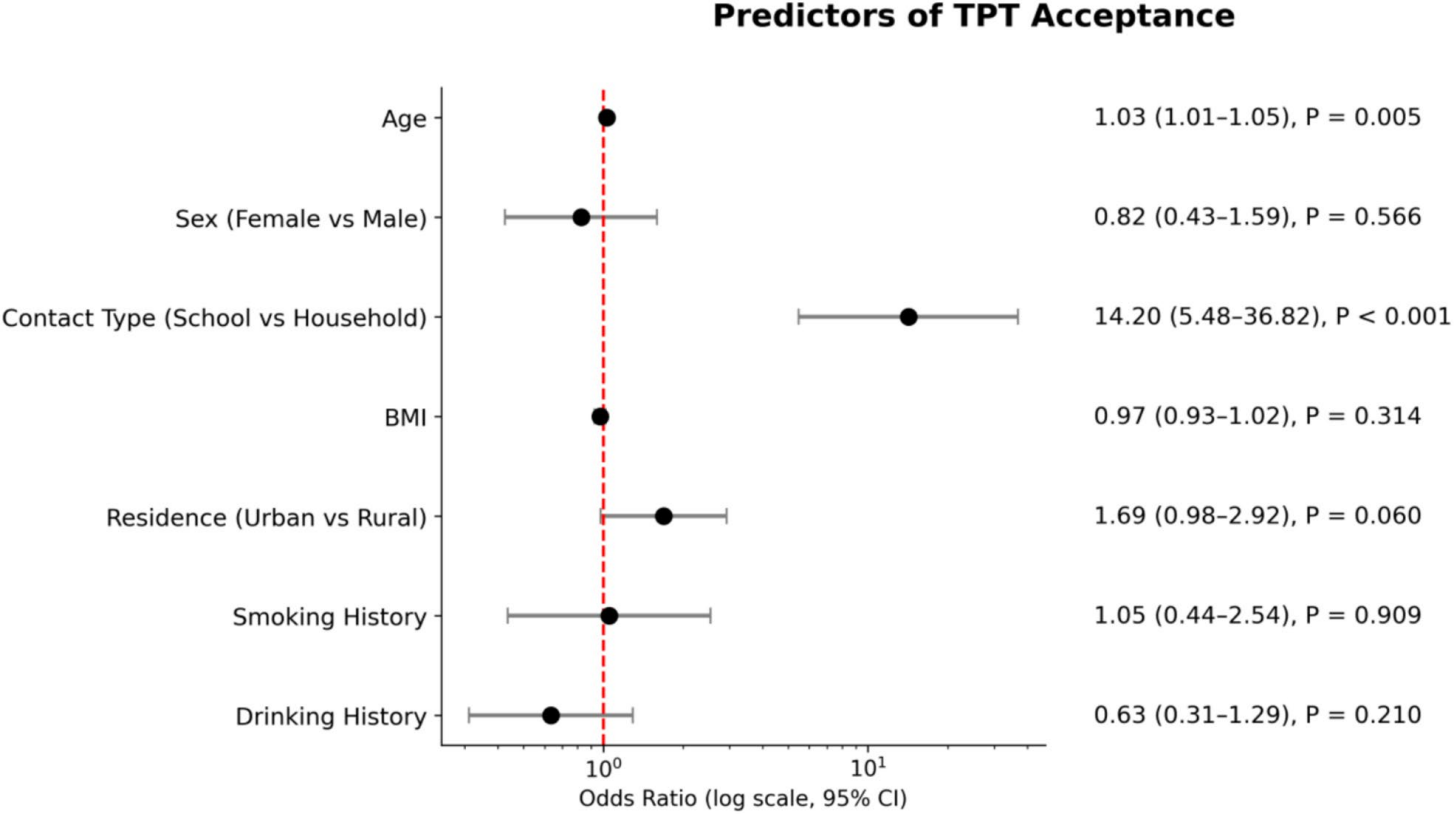
Forest plot of multivariable logistic regression analysis for predictors of TPT acceptance among participants with complete covariate data (*n* = 518).

In multivariable logistic regression among 518 participants with complete covariate data, contact type was the strongest predictor of acceptance. As shown in Figure 2, school contacts had 14.20-fold higher odds of accepting TPT compared with household contacts (adjusted odds ratio 14.20, 95% confidence interval 5.48–36.82, *p* < 0.001). Urban residence showed a trend toward higher acceptance, although the association did not reach statistical significance after adjustment. Although younger participants had higher crude acceptance rates, older age became positively associated with acceptance after adjustment (adjusted odds ratio 1.03 per year, 95% confidence interval 1.01–1.05, *p* = 0.005).

### Treatment completion and predictors

**Figure 3:**
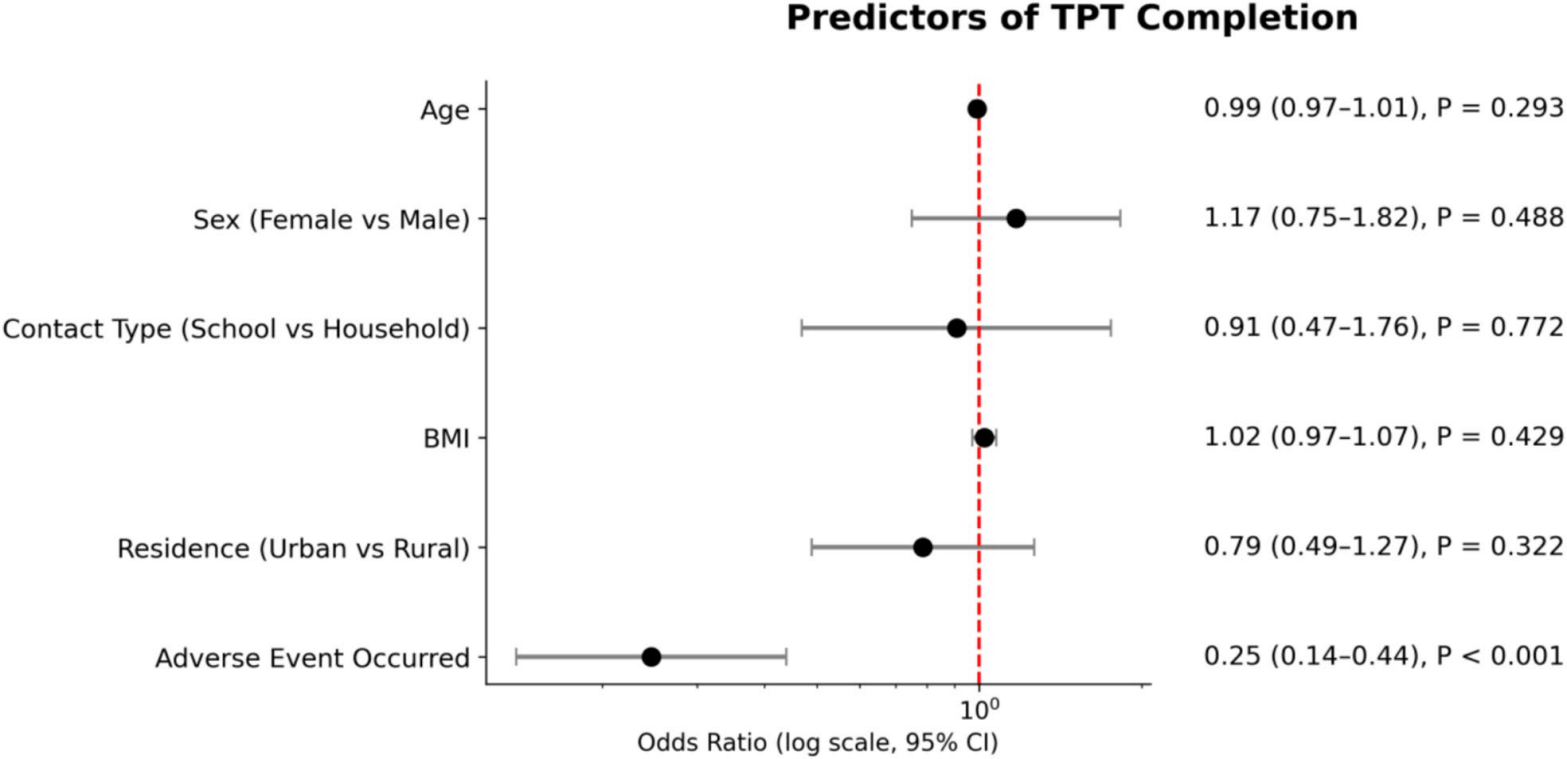
Forest plot of multivariable logistic regression analysis for predictors of TPT completion among treatment initiators with complete covariate data (*n* = 464).

Of the 520 participants who initiated the regimen, 382 participants (73.5%) completed treatment and 138 participants (26.5%) discontinued (Figure 1). In multivariable logistic regression among 464 participants with complete covariate data, adverse events were the only significant factor associated with non-completion. As shown in Figure 3, participants who experienced an adverse event were markedly less likely to complete treatment (adjusted odds ratio 0.25, 95% confidence interval 0.14–0.44, *p* < 0.001). No demographic or clinical factors, including age, sex, contact type, BMI, or residence, were associated with completion.

### Safety and reasons for discontinuation

**Figure 4:**
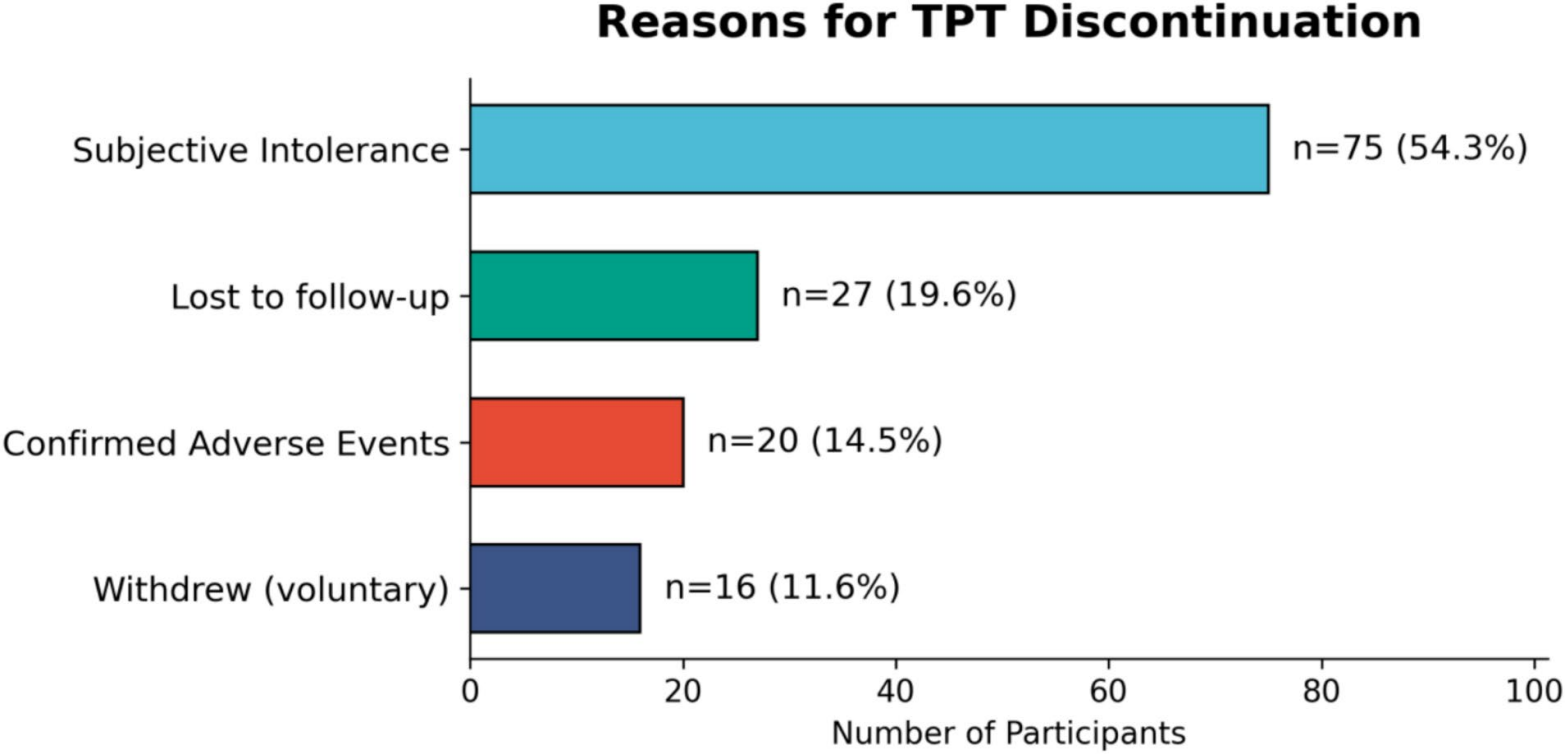
Distribution of specific reasons for treatment discontinuation among those who initiated TPT.

Reasons for treatment discontinuation are shown in Figure 4. Among the 138 participants who discontinued treatment, the most frequently reported reason was subjective intolerance (*n* = 75, 54.3%), followed by loss to follow-up (*n* = 27, 19.6%), clinically confirmed adverse events (*n* = 20, 14.5%), and voluntary withdrawal (*n* = 16, 11.6%). Each participant who discontinued was assigned exactly one primary reason at the time of withdrawal; the categories shown in Figure 4 are therefore mutually exclusive. The most commonly reported symptoms among participants classified as subjective intolerance included fatigue, nausea, headache, and dizziness, none of which were accompanied by laboratory abnormalities meeting prespecified criteria for a clinically significant adverse event. The number of participants discontinuing due to subjective intolerance was substantially higher than those with clinically confirmed adverse events (75 vs. 20).

Adverse events recorded during treatment are shown in Table 2. A total of 56 participants (10.8%) experienced at least one adverse event. Hepatic events were the most common category, accounting for 73.2% of participants with adverse events, with an incidence of 7.9% among treatment initiators. Among the 41 participants with hepatic adverse events, 40 (97.6%) had Grade 1–2 events and 1 (2.4%) had a Grade 3 event; no Grade 4 events occurred. Overall, 15 of 41 participants (36.6%) with hepatic adverse events discontinued treatment. Gastrointestinal and skin events were less frequent but showed high discontinuation proportions within their respective categories, at 40.0 and 50.0%.

**Table 2:** Incidence and spectrum of adverse events (AEs) and their impact on treatment discontinuation among treatment initiators.

Adverse event category	No. of participants with AEs (*N* = 56)	Proportion of participants with AEs (%)	Incidence among treatment initiators (%)	Discontinuation (*n* = 20)	Discontinuation rate within category (%)
Total	56	100.0	10.8	20	35.7
Hepatic events	41	73.2	7.9	15	36.6
Gastrointestinal events	5	8.9	1.0	2	40.0
Skin reactions	4	7.1	0.8	2	50.0
Hematologic abnormalities	3	5.4	0.6	0	0.0
Respiratory symptoms	2	3.6	0.4	1	50.0
Constitutional symptoms	1	1.8	0.2	0	0.0

Each participant with an adverse event was assigned one primary AE category for tabulation. Incidence rates were calculated using all participants who initiated TPT (*N* = 520) as the denominator.

### Sensitivity analysis

To evaluate the differential impact of hepatic and non-hepatic adverse events, we conducted a sensitivity analysis using two logistic regression models (Table 3). In Model A, which included all participants who initiated treatment with complete covariate data (*n* = 468), both hepatic adverse events and non-hepatic adverse events were independently associated with non-completion. In Model B, which excluded participants who experienced hepatic events and retained those with complete covariate data (*n* = 429), non-hepatic adverse events remained significantly associated with lower completion. These results confirm that both hepatic and non-hepatic safety concerns markedly affect adherence to preventive therapy.

**Table 3:** Sensitivity analysis: multivariable logistic regression models evaluating the impact of hepatic and non-hepatic adverse events on TPT completion.

Variables	aOR (95% CI)	*P*-value
Model A: Full sample analysis (*N* = 468^a^)		
Adverse events		
Hepatic AEs (Yes vs. No)	0.30 (0.15–0.59)	**<0.001**
Non-hepatic AEs (Yes vs. No)	0.18 (0.07–0.48)	**<0.001**
Covariates		
Age (per year increase)	0.99 (0.97–1.01)	0.315
Sex (Female vs. Male)	1.13 (0.73–1.74)	0.598
Contact type (School vs. Household)	0.85 (0.44–1.64)	0.629
BMI (per unit increase)	1.02 (0.97–1.07)	0.469
Residence (Urban vs. Rural)	0.74 (0.46–1.19)	0.221
Model B: Excluding participants with hepatic AEs (*N* = 429^b^)		
Adverse events		
Any non-hepatic AE (Yes vs. No)	0.17 (0.06–0.46)	**<0.001**
Covariates		
Age (per year increase)	0.99 (0.97–1.02)	0.587
Sex (Female vs. Male)	1.20 (0.76–1.91)	0.431
Contact type (School vs. Household)	0.86 (0.43–1.74)	0.683
BMI (per unit increase)	1.00 (0.96–1.05)	0.885
Residence (Urban vs. Rural)	0.73 (0.44–1.21)	0.223

aOR, adjusted odds ratio; CI, confidence interval; AE, adverse event; BMI, body mass index; TPT, tuberculosis preventive treatment.

^a^Model A included all participants who initiated TPT with complete covariate data (*N* = 468). Hepatic and non-hepatic AEs were entered as separate variables to assess their independent effects on treatment completion.

^b^Model B excluded participants who experienced hepatic events (*N* = 429) to assess whether non-hepatic AEs remained independently associated with non-completion.

Bold values indicate statistical significance (*P* < 0.05).

## Discussion

In this prospective observational cohort study conducted as a real-world implementation evaluation, we assessed the uptake, completion, and safety of the 3HR regimen among close contacts with latent tuberculosis infection in a high-burden setting in China. Three main findings emerged. First, treatment uptake was high, with 84.3% of eligible contacts initiating TPT. Second, treatment completion declined to 73.5% after initiation, indicating substantial attrition during the post-initiation phase of the TPT cascade. Third, adverse events, particularly hepatic events and subjective intolerance, were the main barriers to treatment persistence. These findings provide practical evidence for optimizing TPT delivery in routine care and are consistent with recent WHO reports showing that losses after treatment initiation remain a major challenge for TPT scale-up (1).

The uptake observed in this study was higher than that reported in many previous studies, in which TPT initiation among eligible contacts often ranged from 40 to 70% (20, 21). The strongest determinant of uptake was contact setting, with school contacts showing substantially higher acceptance than household contacts. This finding supports the value of school-based TPT delivery platforms, where screening, counseling, and follow-up can be implemented in a more structured way (22, 23). By contrast, household contacts may require more active mobilization and clearer risk communication to improve uptake. Although urban residence was associated with higher crude acceptance, this association did not remain statistically significant after adjustment, though it is consistent with previously reported disparities in access to preventive care between urban and rural populations (24, 25). Similarly, younger participants showed higher crude uptake, whereas older age became positively associated with acceptance after adjustment, suggesting that the apparent age difference was influenced by the underlying distribution of contact settings.

Although uptake was high, completion fell to 73.5% among treatment initiators. This level is comparable to that reported in other real-world settings (26), but still indicates meaningful losses after treatment initiation. In the adjusted model, adverse events were the only factor independently associated with non-completion. This suggests that once preventive therapy has been initiated, tolerability becomes the dominant determinant of persistence, outweighing baseline demographic or social characteristics.

A notable finding was the large discrepancy between subjective intolerance and clinically confirmed adverse events. Discontinuation attributed to subjective intolerance was nearly four times more frequent than discontinuation due to clinically confirmed toxicity (75 vs. 20). This suggests that perceived symptom burden may substantially affect adherence even in the absence of laboratory or clinical evidence of significant toxicity. This pattern is consistent with the Health Belief Model, which posits that for asymptomatic individuals, perceived barriers such as somatic discomfort may outweigh the abstract, delayed benefit of disease prevention (27, 28). In addition, nocebo effects, in which negative expectations rather than pharmacological actions trigger adverse symptoms, may have contributed to symptom amplification and premature cessation in this asymptomatic population (29). These findings support the need for structured symptom counseling, anticipatory guidance, and clear triage pathways during TPT delivery (30–32).

Consistent with previous reports, hepatic events were the most frequent adverse events, with an incidence of 7.9% (33, 34). Nearly all graded hepatic events (97.6%) were mild to moderate (Grade 1–2), with only one Grade 3 event and no Grade 4 events. Nevertheless, hepatic toxicity still accounted for a substantial proportion of treatment interruptions and discontinuations. In addition, our sensitivity analyses showed that both hepatic and non-hepatic adverse events independently reduced completion. This indicates that the impact of tolerability is not limited to severe hepatotoxicity, and that non-hepatic symptoms may also reduce persistence in preventive treatment (35, 36).

These findings have several practical implications. School-based delivery models should be further strengthened, given their high acceptance. Household contacts should be prioritized for more intensive mobilization and individualized counseling. Because adverse events were the main determinant of non-completion, early follow-up and structured symptom assessment, particularly during the first month of treatment, may improve persistence. This could include anticipatory guidance regarding common, non-threatening side effects to manage patient expectations (32). The prominence of hepatic events also supports continued liver function monitoring, with risk-stratified approaches potentially considered for participants with symptoms or clinical risk factors (37). Additionally, digital adherence support tools may help clinicians distinguish between subjective distress and clinically actionable events during follow-up (38).

Several limitations should be acknowledged. First, this was a single-center study, which may limit generalizability. Second, participants self-selected whether to initiate TPT, so the findings should be interpreted as reflecting real-world programmatic uptake and completion rather than comparative treatment efficacy. Third, although we adjusted for multiple prespecified covariates, residual confounding cannot be excluded. Fourth, complete-case analysis reduced the effective sample size of the multivariable models. Fifth, LTBI screening relied on TST, which may have limited specificity in this universally BCG-vaccinated population; some participants classified as LTBI-positive may therefore have had false-positive results. Finally, although adverse events were prospectively recorded, we did not conduct qualitative interviews and therefore could not directly examine the mechanisms underlying subjective intolerance (39).

## Conclusion

In conclusion, this real-world implementation evaluation shows that 3HR can achieve high uptake among close contacts with latent tuberculosis infection, particularly in school-based settings, but treatment completion remains limited by both clinically confirmed and patient-perceived adverse events. Optimizing TPT delivery will require stronger household-contact mobilization, structured symptom counseling, and responsive safety monitoring throughout treatment.

## Data Availability

The original contributions presented in the study are included in the article/supplementary material, further inquiries can be directed to the corresponding authors.
